# Reply to Fernandez-Vigo et al. Comment on “Torrellas et al. Effectiveness, Safety and Choroidal Changes of a Fovea-Sparing Technique for the Treatment of Chronic Central Serous Chorioretinopathy with Yellow Subthreshold Laser. *J. Clin. Med.* 2023, *12*, 1127”

**DOI:** 10.3390/jcm12175440

**Published:** 2023-08-22

**Authors:** Beatriz Torrellas, Alejandro Filloy, Lihteh Wu, Jay Chhablani, Pedro Romero-Aroca

**Affiliations:** 1 Ophtalmology Department, Joan XXIII University Hospital, 43007 Tarragona, Spain; 2 Institut d’Investigació Sanitaria Pere Virgili (IISPV), Rovira & Virgili University, 43204 Reus, Spain; 3 Clínica Oftalmològica de Tarragona (COT), 43001 Tarragona, Spain; 4 Asociados de Mácula, Vítreo y Retina de Costa Rica, San José 10102, Costa Rica; 5 UPMC Eye Center, University of Pittsburgh, Pittsburgh, PA 15213, USA; 6 Ophthalmology Department, University Hospital Sant Joan de Reus, 43204 Reus, Spain

We thank the comment written by Fernández-Vigo et al. [1] on our study [2]. Their points are of high interest, and we would like to make further clarification. 

First, we considered a variation in the best corrected visual acuity (BCVA) to be any variation larger or equal than 0.2 on a decimal scale and stabilization when the BCVA stood within this range. Also, we are aware that the BCVA measured using logMAR or ETDRS charts is more accurate than the decimal scale. The reason why we chose to use the decimal scale was because of the logistics of our hospital. This is one of the main reasons why the study of BCVA was not the main purpose of study, although it helps to understand the evolution of the visual function of the patient. We also did not convert BCVA from the decimal scale to logMAR or ETDRS because it would be considered a confusion factor [3]. Our goal is to continue the study by changing the Snellen charts to logMAR ones and present our new data in a future study.

To respond to the second and third points, we are providing further data: the mean energy employed after titration was 357.1 ± 70.4 mW (range 250 to 600). The mean number of spots used was 598.3 ± 233.1 (range 270 to 1430) with a size of 160 microns. 

In regard to the fourth point, we have not yet recorded the presence, height, or size of serous pigmentary epithelium detachment (PED). This is a limitation of our study, although it is a fairly interesting point for future studies.

Fifth, the measurement of subretinal fluid (SRF), as well as the remaining measurements, were performed by two different observers in a masked way in every visit. Meaning that every test (Swept-Source Optical Coherence Tomography (SS-OCT), autofluorescence, and fluorescein angiography tests) was analyzed by one of the two retinal specialists without knowledge of the other. In regard to the masked fashion that Fernández-Vigo et al. suggest, one of the retinal specialists knew if the images corresponded to the visit after treatment and the other retinal specialist did not. Nevertheless, all measurements had a strong inter-observer reproducibility (ICC of 0.99), meaning that an involuntary bias was probably avoided.

In reference to the sixth point, the recurrence of SRF after subthreshold laser treatment (STL) was defined as the presence of SRF after complete resolution documented in SS-OCT, while the persistence of SRF after STL without complete resolution was not considered a recurrence but an unresolved case. In relation to this, it seems to be an association with a higher chronicity and the need of retreatment, but more studies with a higher sample size are needed to validate this association. In relation to the retreatment of STL, patients retreated received the same parameters as in their first session.

We hope these clarifications help to improve the design and results of studies focused on central serous chorioretinopathy treatment.
